# Carcinoid tumor of the verumontanum (colliculus seminalis) of the prostatic urethra with a coexisting prostatic adenocarcinoma: a case report

**DOI:** 10.1186/1752-1947-4-16

**Published:** 2010-01-20

**Authors:** Maxwell Smith, M Scott Lucia, Priya N Werahera, Francisco G La Rosa

**Affiliations:** 1 University of Colorado Denver, Department of Pathology, Aurora, Colorado 80045-0508, USA

## Abstract

**Introduction:**

Urethral carcinoid tumors are very rare tumors with only four cases described in the literature.

**Case presentation:**

We present the case of a 61-year-old man with a primary carcinoid tumor of the verumontanum (colliculis seminalis) portion of the prostatic urethra with a coexisting prostatic adenocarcinoma. In addition to whole mount hematoxylin and eosin staining, special immunoperoxidase staining specific for chromogranin A, neuron specific enolase, synaptophysin, pan-cytokeratin and PSA, and a special combined staining for racemase (α-methyl CoA) antigen and p63 antigen were performed. A review of the literature is included.

A single focus of invasive prostatic adenocarcinoma was identified in the periphery of the mid-left, posterior quadrant of the prostate. Approximately 17 mm from this adenocarcinoma, within the verumontanum of the prostatic urethra, there was a 3 mm maximal dimension carcinoid tumor.

**Conclusion:**

Based on different histological features and antigenic profiles, we concluded that the two tumors were distinct.

## Introduction

Carcinoid tumors of the urethra are exceedingly rare neoplasms, with only four cases reported in the literature [1-4]. Given this small number, their clinical presentation, course and prognosis are difficult to elucidate.

We report the first case of a primary carcinoid tumor within the verumontanum of the prostatic urethra in a 61 year-old man who underwent radical prostatectomy for adenocarcinoma of the prostate. We reviewed the literature for both primary carcinoid tumors of the urethra and for synchronous urethral carcinoid tumors and prostatic adenocarcinoma.

## Case presentation

Our patient is a 61-year-old Caucasian man who originally presented with urinary obstructive symptoms and a serum prostate specific antigen (PSA) level of 1.5 ng/mL six years before prostatectomy. The obstructive process was treated conservatively, with observation and symptomatics. Over the following three years, PSA of our patient continued to rise, prompting multiple sets of prostatic biopsies that were negative for carcinoma. Ultrasound showed a moderately enlarged nodular prostate consistent with benign prostatic hyperplasia. A few weeks before prostatectomy, his serum PSA increased up to 4.7 ng/mL, at which time additional core biopsies were performed. A single focus of Gleason grade 3+3 (score = 6) prostatic adenocarcinoma was identified, involving 5% of only one of the tissue cores submitted. Our patient was subject to undergo a radical prostatectomy. A conventional suprapubic, radical prostatectomy on our patient was performed. The prostate was submitted for whole-mount processing. Gross examination revealed a 32 gm prostate measuring 4.2 cm apex-base, 4 cm wide, and 3.5 cm antero-posterior. The attached seminal vesicles were up to 2.4 cm in length and 0.4 cm average diameter. Also submitted were dissections of the right and left pelvic lymph nodes. The prostate was fixed in formalin, serially sectioned in 5 mm intervals from apex to the base and paraffin embedded. Histological sections, 5 μm-thick, were cut from the anterior face of each paraffin block, mounted on 2 × 3-inch glass slides and stained with conventional hematoxylin and eosin. Immunoperoxidase staining specific for chromogranin A, neuron specific enolase, synaptophysin, pan-cytokeratin and PSA, and a special combined staining for racemase [α-methyl CoA] antigen and p63 antigen were performed.

Digital microscopic pictures were obtained during different magnifications using an Olympus BX51 microscope (Olympus, Japan) and a Macrofire digital camera (Optronics, Goleta, CA) connected though a firewire to a Dell Optiplex 745 desktop computer (Dell, USA). Low power images from whole mount slides were digitally scanned using an Aperio ScanScope Model T3 (Aperio Technologies, Inc., Vista, CA, USA) and captured using the ImageScope software version 9.x (Aperio Technologies, Inc., Vista, CA, USA). All images were optimized using Adobe Photoshop CS2 software (San Diego, CA).

**Figure 1:**
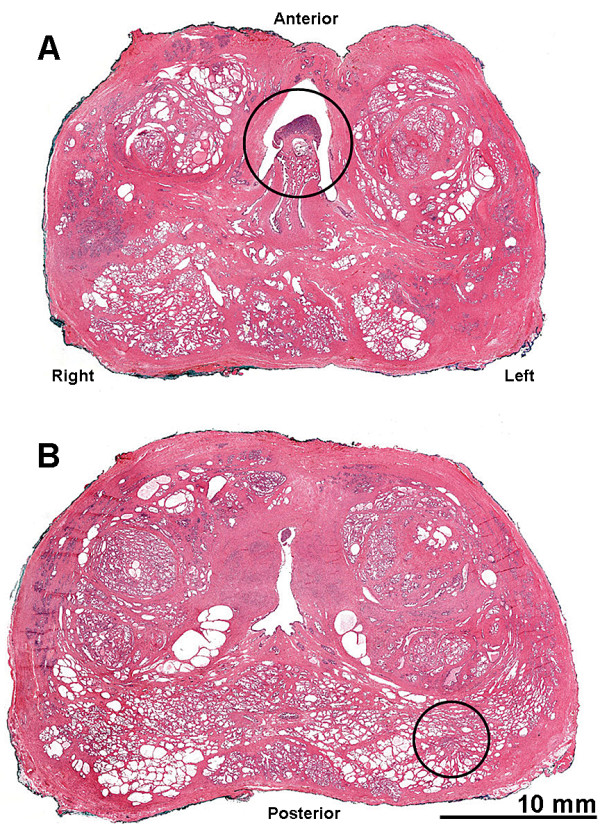
**Location of (A) the carcinoid tumor in the verumontanum of the prostatic urethra; and (B) the prostatic adenocarcinoma within the prostate at the right posterior lobe of the prostate**. These consecutive whole prostatectomy slices were sectioned 5 mm apart and the tumors appear to be located at approximately 17 mm from each other. Hematoxylin-eosin staining (scanned slides with Aperio system).

**Figure 2:**
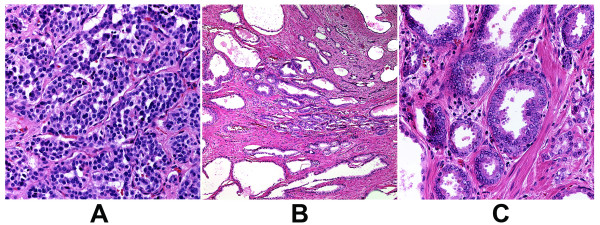
**(A) Carcinoid tumor (40× objective), (B) and C) prostatic adenocarcinoma Gleason grade 3 + 3 (sum = 6) (10× & 40× objectives)**. Hematoxylin-eosin staining.

A single focus of invasive, variably spaced, small atypical acinar glands without basal cells was identified in the periphery of the mid-left, posterior quadrant of the prostate, involving less than 5% of the prostatic tissue (Figure 1). The cytologic features were consistent with prostatic adenocarcinoma and included enlarged and overlapping nuclei with prominent and peripherally marginated nucleoli (Figures 2Band 2C). The tumor from our patient was organ confined, without angiolymphatic, perineural, extracapsular or seminal vesicle invasion, and with negative surgical margins. Additional histopathology included focal high grade prostatic intraepithelial neoplasia, benign prostatic hyperplasia, focal acute and chronic inflammation, focal glandular atrophy and basal cell hyperplasia. The two right and two left pelvic lymph nodes were without evidence of malignancy.

**Figure 3:**
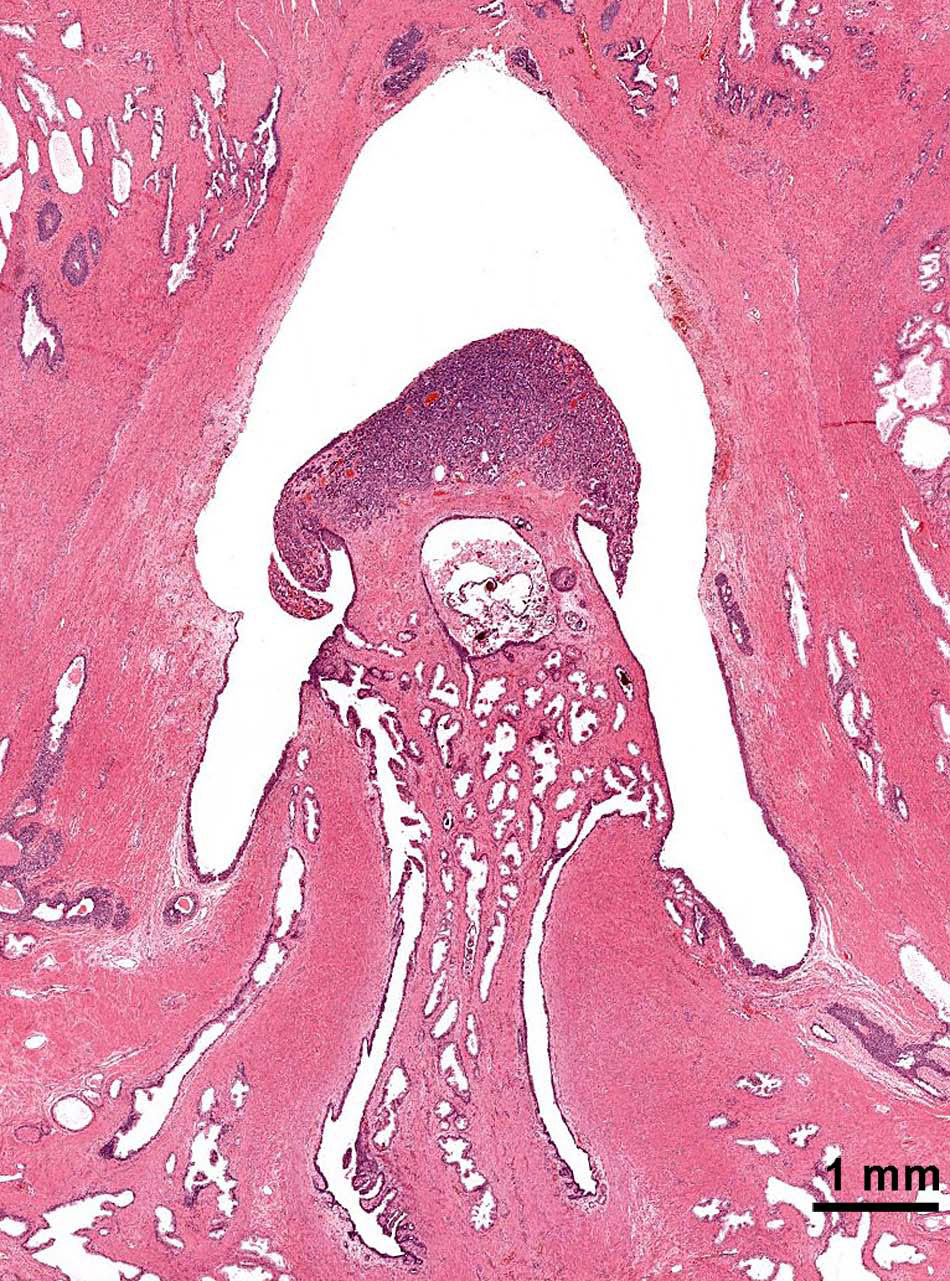
**Carcinoid tumor of the veramontanum (colliculus seminalis) of the prostatic urethra, hematoxylin-eosin staining (2× objective)**.

**Figure 4:**
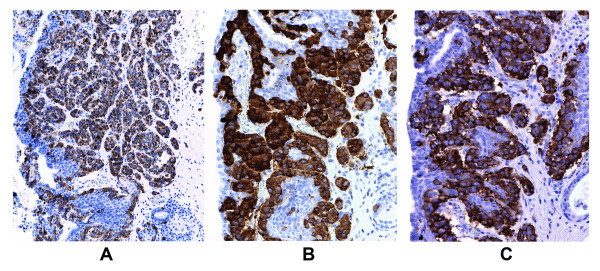
**Immunoperoxidase staining for chromogranin A (20×) (A), neuron specific enolase (B) and synaptophysin (C) of the carcinoid tumor cells (40× objective)**.

Approximately 17 mm from the invasive adenocarcinoma, within the verumontanum of the prostatic urethra of our patient, there was a 3 mm maximal dimension tumor with markedly different morphology. From low microscopic power, the tumor of our patient appeared well circumscribed with pushing, rather than invasive, margins (Figure 3). Higher power examination revealed cells forming nested, acinar and trabecular architecture with small uniform nuclei, a granular "salt and pepper" chromatin pattern, and moderate amounts of granular cytoplasm (Figure 2A). Mitotic figures, necrosis, and angiolymphatic invasion were not identified. Immunoperoxidase staining for chromogranin A, neuron specific enolase, and synaptophysin were positive in nearly 100% of the urethral tumor cells, showing a granular cytoplasmic distribution (Figure 4). These cells were negative for pan-cytokeratin and PSA. Immunoperoxidase staining for PSA was positive in the prostatic adenocarcinoma cells, which also were positive for racemase (α-methyl CoA) antigen and negative for p63 antigen. Based on this pattern of reactivity, we concluded that the two tumors were distinct and a diagnosis of carcinoid tumor was made on the urethral tumor of our patient. An electron microscopy was attempted on our patient for neurosecretory granules in the urethral tumor, but it was unsuccessful due to formalin fixation artifact.

## Discussion

Carcinoid tumors of the urethra are exceedingly rare neoplasms. Using Pubmed and the search words "urethra" and "carcinoid" only four articles are found, each reporting a single additional case of a urethral carcinoid tumor [1-4]. The first case of urethral carcinoid, reported by Sylora et al. in 1975, involved an 8 mm lesion of the penile urethra in a 47-year-old man. Including our case, urethral carcinoid tumors primarily appear on men (men:women 4:1), with an average age of 47 years (range 39-60 years), and an average size of 5.6 mm (range 3-8 mm). Urethral carcinoids appear to be more common in the penile urethra with three of the four male cases occurring in that location. The case in the woman occurred at the urethral orifice. All cases, including our case, showed a similar histology characterized by sheets, acini, trabeculae, and nests of small uniform cells with a coarse nuclear chromatin pattern. Neuroendocrine differentiation of the tumor cells was confirmed in each instance by immunohistochemical analysis for neuron-specific enolase, chromogranin A, and/or synaptophysin and/or electron microscopy studies showing neurosecretory granules.

While concurrent prostatic adenocarcinoma and urethral carcinoid tumor have yet to be reported, concurrent prostatic adenocarcinoma and prostatic carcinoid tumors are rarely reported [5,6]. Because prostatic adenocarcinoma may undergo neuroendocrine differentiation, with prognostic and treatment implications, its distinction from primary carcinoid is imperative. A combination of histologic, immunohistochemical, and geographical differences between the two tumors in question, as seen in our present case, are used to make the diagnosis. Immunological markers to identify a prostatic gland origin of the tumor included PSA, combined racemase (α-methyl CoA) and p63 antigen and pan-cytokeratin. Markers to identify the neuro-endocrine origin of the second tumor include chromogranin, synaptophysin and neurone specific enolase.

## Conclusion

Our case is unique in many ways. It is the first case of a patient with a primary carcinoid tumor in the prostatic urethra, and more specifically within the verumontanum. Secondly, it represents the oldest patient reported with a urethral carcinoid (61 years old). It also represents the first case of a concurrent urethral carcinoid with a primary adenocarcinoma of the prostate.

## Abbreviations

PSA: Prostate specific antigen.

## Consent

Written informed consent was obtained from our patient for publication of this case report and accompanying images. A copy of the written consent is available for review by the Editor-in-Chief of this journal.

## Competing interests

The authors declare that they have no competing interests.

## Authors' contributions

MS was the major contributor in writing the manuscript. MSL and PNW reviewed and discussed the manuscript. FGLR performed the original histological examination of the prostate, interpreted and diagnosed the pathology findings, prepared the figures and did the literature review. All authors read and approved the final manuscript.
